# Distinct Roles of Two DNA Methyltransferases from Cryphonectria parasitica in Fungal Virulence, Responses to Hypovirus Infection, and Viral Clearance

**DOI:** 10.1128/mBio.02890-20

**Published:** 2021-02-09

**Authors:** Yo-Han Ko, Kum-Kang So, Jeesun Chun, Dae-Hyuk Kim

**Affiliations:** a Department of Molecular Biology, Institute for Molecular Biology and Genetics, Jeonbuk National University, Jeonju, Chonbuk, South Korea; b Department of Bioactive Material Science, Institute for Molecular Biology and Genetics, Jeonbuk National University, Jeonju, Chonbuk, South Korea; University of British Columbia

**Keywords:** *Cryphonectria parasitica*, DNA methyltransferase, fungal growth and development, hypovirulence, viral clearance

## Abstract

Two DNA methyltransferase (DNMTase) genes from Cryphonectria parasitica have been previously identified as *CpDmt1* and *CpDmt2*, which are orthologous to *rid* and *dim-2* of Neurospora crassa, respectively. While global changes in DNA methylation have been associated with fungal sectorization and *CpDmt1* but not *CpDmt2* has been implicated in the sporadic sectorization, the present study continues to investigate the biological functions of both DNMTase genes. Transcription of both DNMTases is regulated in response to infection with the *Cryphonectria* hypovirus 1 (CHV1-EP713). *CpDmt1* is upregulated and *CpDmt2* is downregulated by CHV1 infection. Conidium production and response to heat stress are affected only by mutation of *CpDmt1*, not by *CpDmt2* mutation. Significant changes in virulence are observed in opposite directions; i.e., the *CpDmt1*-null mutant is hypervirulent, while the *CpDmt2*-null mutant is hypovirulent. Compared to the CHV1-infected wild type, CHV1-transferred single and double mutants show severe growth retardation: the colony size is less than 10% that of the parental virus-free null mutants, and their titers of transferred CHV1 are higher than that of the wild type, implying that no defect in viral replication occurs. However, as cultivation proceeds, spontaneous viral clearance is observed in hypovirus-infected colonies of the null mutants, which has never been reported in this fungus-virus interaction. This study demonstrates that both DNMTases are significant factors in fungal development and virulence. Each fungal DNMTase affects fungal biology in both common and separate ways. In addition, both genes are essential to the antiviral responses, including viral clearance which depends on their mutations.

## INTRODUCTION

The introduction of Cryphonectria parasitica, an ascomycete filamentous fungus, devastated chestnut forests in North America at the beginning of the twentieth century. However, infection by the single-stranded, previously classified as double-stranded RNA hypovirus *Cryphonectria* hypovirus 1 (CHV1) results in attenuation of virulence, known as hypovirulence, in C. parasitica, as well as other associated symptoms such as altered metabolism, retarded development, and reduced sporulation (1). Moreover, CHV1 can be naturally transferred to other fungal hosts during hyphal fusion, converting virus-free virulent strains into virus-infected hypovirulent strains, resulting in the protection of chestnut trees from detrimental blight disease. This virus-fungus-plant interaction has been a successful example of naturally occurring biological control (1–3). Considering the difficulties and limited means available in the control of fungal disease, this type of biocontrol using hypovirulent mycovirus, known as virocontrol, has attracted considerable attention as an alternative method of controlling plant-pathogenic fungi (4). Since molecular techniques, including transformation (5), gene replacement (5), the use of cDNA-infectious copies of the hypovirus (6), and transcriptomic (7, 8) and proteomic analyses (9), have been well established in *C. parasitica*, studies of functional genomics (10, 11), signal transduction (12, 13), gene silencing (14), and epigenetics (15–17) have been successfully conducted. Therefore, *C. parasitica* and its hypoviruses have come to serve as well-known model systems for research on fungus-mycovirus interactions (18).

Recent studies of fungal gene regulation have revealed the presence of epigenetic mechanisms such as chromatin modification, DNA methylation, and noncoding RNAs (19–22). These epigenetic changes have been implicated not only in regulating the expression of specific fungal genes but also in driving global gene expression patterns. Among these mechanisms, it has been suggested that DNA methylation in fungi, which exists at various levels, is involved in a broad spectrum of biological processes, which is mainly attributable to genome defense and developmental processes such as colonial growth, sporulation, sexual reproduction, toxin production, stress responses, and virulence (23–26). Recently, the characteristics of genome-wide DNA methylation in *C. parasitica* have been described, with dramatic changes in DNA methylation being observed in mutant progeny that have undergone severe morphogenic changes such as sectorization (15). However, at this time, little is known about the role of DNA methylation-based epigenetic regulation in controlling gene expression in multicellular fungi.

DNA methylation occurs at selected cytosine bases of eukaryotic DNA, which are converted to 5-methylcytosine by DNA methyltransferase (DNMTase). For DNMTases, “maintenance” and “*de novo*” methylation are the two major types of activities (27). While maintenance methylation occurs after DNA replication at hemimethylated motifs, methylation of previously unmethylated cytosines is known as *de novo* methylation. On the basis of sequence similarity and function, eukaryotic DNMTases have been grouped into six subfamilies: DNMT1/MET1, DNMT2/PMT1, DNMT3/DRM, DNMT4/RID, DNMT5, and DNMT6 (28). Among these groups, leaving aside the presence of tRNA methyltransferase DNMT2, ascomycete fungal genomes predominantly harbor DIM-2, RID, and DNMT5 or DIM-2 and RID (29). Domain searching followed by phylogenic analysis of DNA methylase domain-containing proteins of *C. parasitica* draft genome sequence (http://genome.jgi-psf.org/Crypa2/Crypa2.home.html) reveals the presence of putative DIM-2, RID, and DNMT5 (29, 30).

In an earlier study, we identified two representative DNMTases in *C. parasitica* and constructed null mutant strains for each DNMTase to enable phenocopying of sectorization (15). However, further studies of other biological characteristics were required. In the present study, taking advantage of the availability of single mutants and the construction of new double mutants, we examine the biological functions of each DNMTase gene both alone and in combination. We observe novel changes in pathogenicity and the response to hypoviral infection.

## RESULTS

### Characteristics of the DNMTase genes *CpDmt1* and *CpDmt2* and their expression.

Sequence analysis revealed the presence of characteristic domains of DNMTases, while phylogenetic analysis of their deduced amino acid sequences indicated that *C. parasitica Dmt1* (*CpDmt1*) and *CpDmt2* genes (GenBank accession no. MF000328 and MF000329, respectively) are orthologs of the repeat-induced point mutation defective (*rid*) and defective in DNA methylation (*dim-2*) genes of Neurospora crassa, respectively.

**FIG 1:**
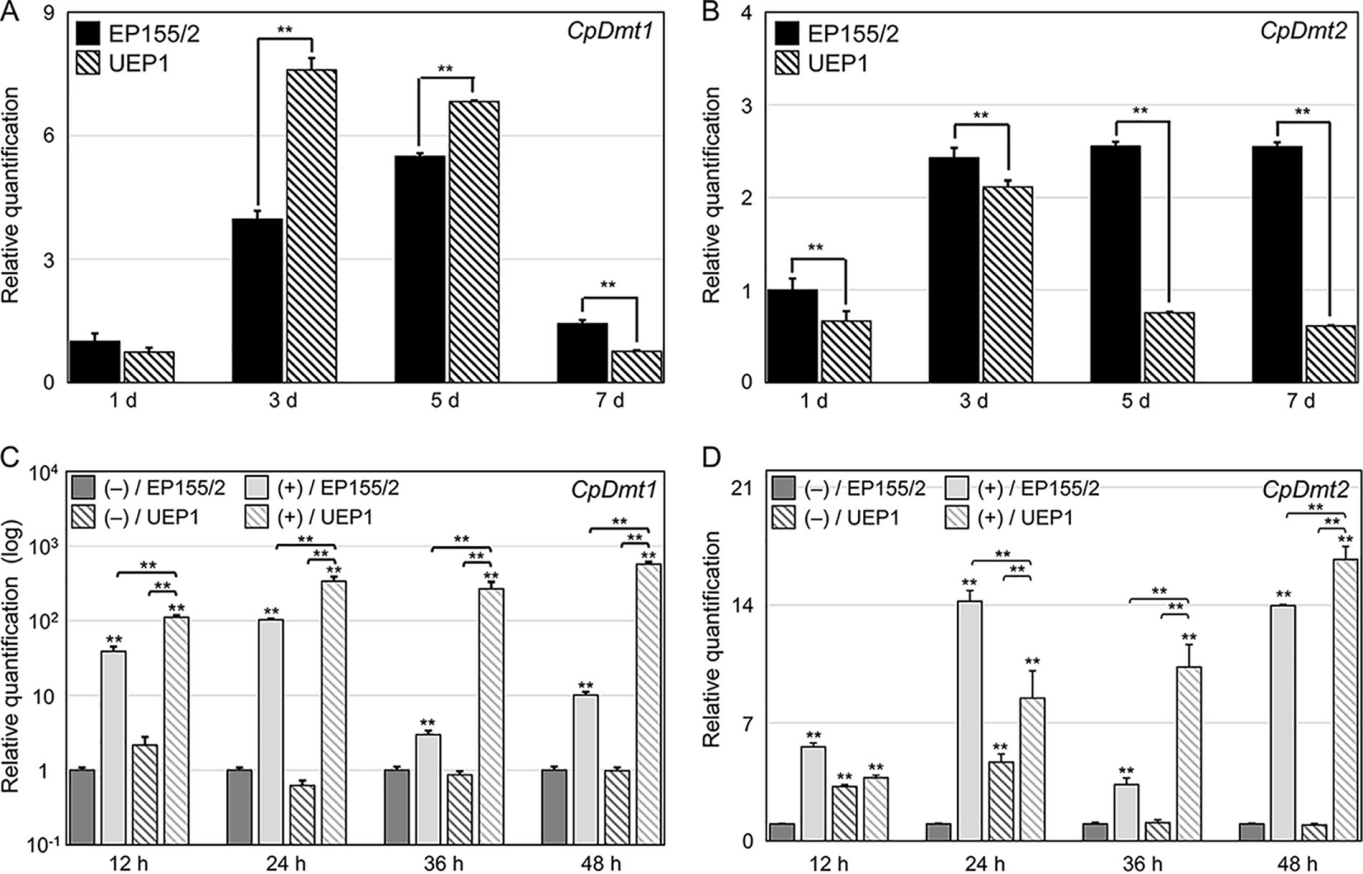
Expression analyses of *CpDmt1* and *CpDmt2.* (A and B) qRT-PCR results of expression levels of *CpDmt1* and *CpDmt2* during cultivation in standard liquid EP complete medium. (C and D) qRT-PCR results of the expression levels of *CpDmt1* and *CpDmt2* during cultivation on tannic acid (TA)-supplemented medium. The numbers of days (d) and hours (h) in liquid culture and TA-supplemented medium are shown below the bars in panels A to D. The strains are indicated in the top leftmost corner of each panel, and media with (+) or without (–) TA supplementation are indicated. At least three individual experiments were performed. Values are means plus standard deviations (SD) (error bars). Student’s *t* test was used to compare data between two groups (**. *P* < 0.01). Strains used were virus-free wild-type EP155/2 and its isogenic CHV1-infected UEP1 strain.

To analyze how CHV1 affects the expression of DNMTase genes, we examined the accumulation of transcripts of each of the DNMTase genes in both the wild-type EP155/2 strain and its isogenic CHV1-EP713-infected hypovirulent strain UEP1 under standard liquid-culture conditions using real-time reverse transcription-PCR (RT-PCR) (Fig. 1A and B). The accumulation of *CpDmt1* transcripts was significantly elevated in 3-day cultures of the isogenic CHV1-infected UEP1 strain compared to the wild type; after remaining constant for up to 5 days of cultivation, it then decreased to a level lower than that of the wild type, indicating that CHV1 infection significantly altered the accumulation of *CpDmt1* transcripts in *C. parasitica* (Fig. 1A). The accumulation of *CpDmt2* transcripts was affected by CHV1 infection, but it was significantly downregulated in the UEP1 cultures compared to the wild type (Fig. 1B). Therefore, while the expression of both *CpDmt1* and *CpDmt2* genes was affected by the presence of the hypovirus CHV1, the effect operated in opposite directions. Transcriptional changes in DNMTases were investigated when the cultures were transferred onto solid medium supplemented with tannic acid (TA), which is abundant in the bark of chestnut tree and is thus considered to be one of the best methods for measuring the virulence of *C. parasitica* (31) (Fig. 1C and D). While significant induction of transcript levels was observed in both DNMTase genes as a result of supplementation with TA, it was particularly dramatic in the *CpDmt1* gene compared to the *CpDmt2* gene, with a >10- to >100-fold upregulation (Fig. 1C). Significant differences in upregulation were observed between TA-induced CHV1-free EP155/2 and TA-induced CHV1-infected UEP1, suggesting that CHV1 also upregulated the *CpDmt1* transcript level under TA-supplemented conditions. Fold changes in *CpDmt2* upregulation were less dramatic than those in the case of *CpDmt1*, while the effect of CHV1 on the accumulation of *CpDmt2* transcript on the TA-supplemented solid medium differed from that in standard liquid medium (Fig. 1D).

### Construction of the double-knockout mutants.

For combined functional analysis of the *CpDmt1* and *CpDmt2* genes, a double-knockout (*CpDmt1* and *CpDmt2*) mutant was constructed using previously described *CpDmt1*-null (TdDMT1 [transformant-deleted DNMTase 1 gene]) and *CpDmt2*-null mutants (TdDMT2 [transformant-deleted DNMTase 2 gene]) as a host strain through integrative transformation-mediated gene replacement (15). A total of 54 stable transformants using the *CpDmt2*-null vector (pDGDMT2, pDDMT2::G418) and TdDMT1 as the host strain were selected, and single spores were isolated. The resulting single-spore transformants were screened using PCR as described previously (10), with two pairs of outer gene-specific and inner *G418* primers (primers 5 and 4 and primers 3 and 6 in Fig. S1A in the supplemental material). Two transformants showed 5,128-bp PCR amplicons, which corresponded to the expected disrupted allele size of the *CpDmt2* gene (Fig. S1A). Southern blot analysis using XhoI digestion and a probe confirmed the replacement of the wild-type allele with the disrupted allele in these two transformants (data not shown), which were accordingly designated TdDMT1/2-1 and -2. Likewise, a total of 83 stable transformants using the *CpDmt1*-null vector (pDGDMT1, pDDMT1::G418) and TdDMT2 as a host strain were screened through PCR as described previously with two pairs of outer gene-specific and inner *G418* primers (primers 1 and 4 and primers 3 and 2 in Fig. S1B). Two transformants showed 5,129-bp PCR amplicons, corresponding to the expected disrupted alleles of the *CpDmt1* gene (Fig. S1B). Southern blot analysis using HindIII digestion and a probe confirmed replacement of the wild-type allele with the disrupted allele in these two transformants (data not shown), which were accordingly designated TdDMT2/1-1 and -2.

10.1128/mBio.02890-20.1 FIG S1 Schematic diagrams of restriction map and gene replacement for double mutants based on *CpDmt1*-null (TdDMT1) (A) and *CpDmt2*-null (TdDMT2) (B) mutants. (A) Restriction map of *CpDmt1* genomic region and *CpDmt1*-null mutation with the desired replacement of *CpDmt1* is presented with the expected changes in size of replacement fragments (left panel). PCR analysis of expected *CpDmt1*-null mutants is shown (right panel). PCR amplicons marked by asterisks, arrows, and arrowheads indicate wild-type allele of *CpDmt1* and null alleles of *CpDmt1* and *CpDmt2*, respectively. (B) Restriction map of *CpDmt2* genomic region and *CpDmt2*-null mutation with desired replacement of *CpDmt2* are presented with expected changes in size of replacement fragments (left panel). PCR analysis of expected *CpDmt1*-null mutants is shown (right panel). PCR amplicons marked by double asterisks, arrows, and arrowheads indicate wild-type allele of *CpDmt2* and null alleles of *CpDmt1* and *CpDmt2*, respectively. *hph*^R^ and *G418*, indicated by dashed box, represent hygromycin B resistance cassette and Geneticin resistance cassette, respectively. H and X represent restriction endonucleases HindIII and XhoI, respectively. Primer pairs are indicated by arrows, while the probes are indicated by black lines below the corresponding restriction map. Strains are indicated at top of each lane, with lane M containing a 10-kb size marker. Download FIG S1, TIF file, 2.9 MB. Copyright © 2021 Ko et al. 2021 Ko et al. https://creativecommons.org/licenses/by/4.0/ This is an open-access article distributed under the terms of the Creative Commons Attribution 4.0 International license.

### Morphological characteristics of the DNMTase mutants and their responses to stress conditions.

**FIG 2:**
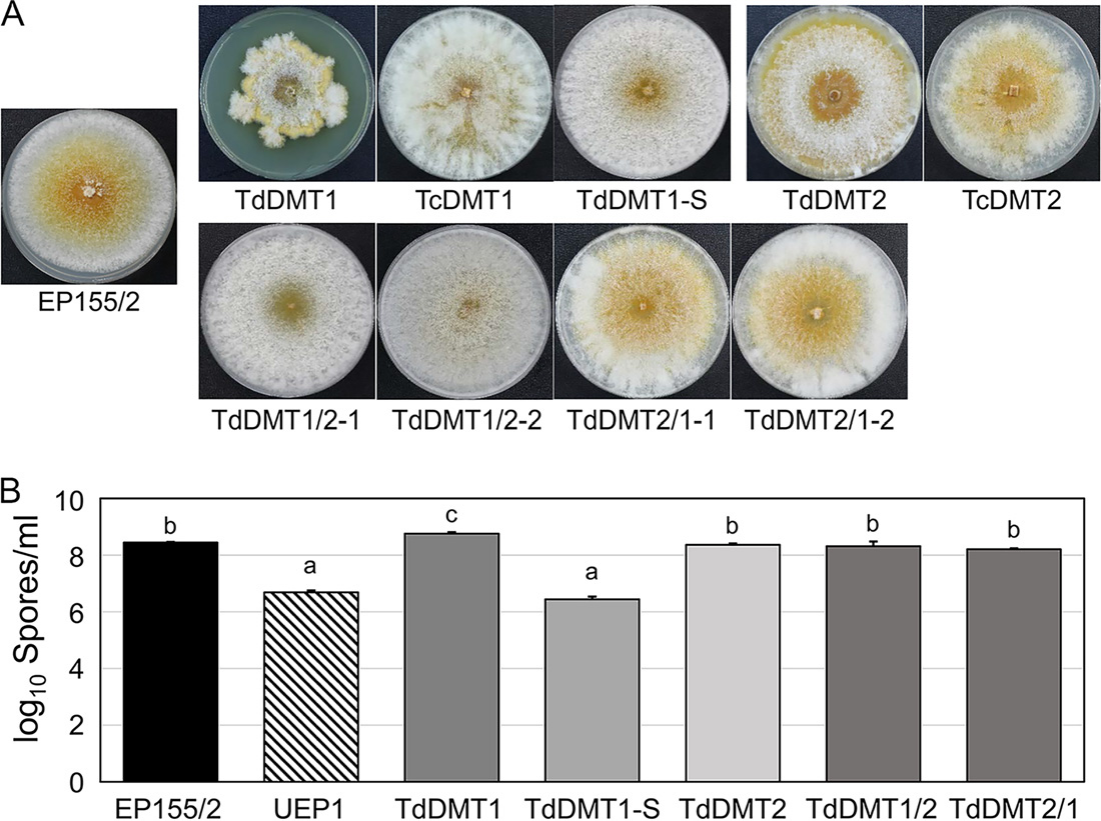
Colony morphology and conidium production of the mutant strains. (A) Colonies are shown after 14 days of cultivation on PDAmb. (B) Analysis of conidium production. Strains are indicated below the photographs and include the wild-type EP155/2, *CpDmt1*-null mutant (TdDMT1), a sectored progeny of TdDMT1 (TdDMT1-S), *CpDmt2*-null mutant (TdDMT2), complementing strains of TdDMT1 and TdDMT2 (TcDMT1 and TcDMT2, respectively), two independently isolated double mutants constructed through *CpDmt2*-null mutation from TdDMT1 (TdDMT1/2-1 and -2), and two independently isolated double mutants constructed through *CpDmt1*-null mutation from TdDMT2 (TdDMT2/1-1 and -2). The mean comparisons of conidia harvested from at least three individual experiments were analyzed using one-way ANOVA with Duncan’s method. The means with a common letter are not significantly different (**, *P* < 0.01). Values are means plus SD (error bars).

Phenotypic changes in the colony morphology of single DNMTase mutants have been previously reported, with the *CpDmt1*-null mutant (TdDMT1) showing retarded colonial growth with sporadic sectorization; that is, as culture was prolonged, TdDMT1 began to show sporadic sectorization with fluffy mycelial growth. This differed from the colony morphology of parental TdDMT1, while single-spore cultures of these fast-growing sectors maintained the characteristics of active mycelial growth, with reduced conidiation and restricted pigmentation, indicating the stable inheritance of the sectored phenotype and designated TdDMT1-S. The *CpDmt2*-null mutant (TdDMT2) showed a similar growth rate to that of the wild-type strain, with the disappearance of peripheral aerial hyphae concurrent with the uncovering of pigmented spore-bearing structures (15) (Fig. 2A). Whole-genome sequencing of TdDMT1 and TdDMT1-S revealed no difference in the genome sequences of either of the mutant strains from the reference genome sequence (http://genome.jgi-psf.org/Crypa2/Crypa2.home.html) other than the replaced allele of the *CpDmt1* gene; thus, no sequence deviation existed between TdDMT1 and TdDMT1-S (BioProject accession no. PRJNA657707). As with other sectored progenies of mutants in the cell wall integrity signal transduction pathway (11, 13), TdDMT1-S was therefore not due to a simple second mutation but rather to epigenetic changes, as previously discussed (11, 13, 15). Two double-knockout mutants (TdDMT1/2-1 and -2) from a single-knockout parent (TdDMT1) showed vigorous mycelial growth compared to that of the parental strain, TdDMT1; however, their pigmentation was more restricted and less extensive than in the parental TdDMT1, which was similar to the wild type (Fig. 2A, TdDMT1 [top row; first three plates] and double mutants [bottom row]). The two double-knockout mutants (TdDMT2/1-1 and -2) constructed from the other parental single-knockout mutant (TdDMT2) showed growth phenotypes similar to that of the parental TdDMT2 mutant, aside from the presence of abundant peripheral aerial hyphae with diffused pigmentation (Fig. 2A; TdDMT2 [top row; final two plates] and double mutants [bottom row]). Regardless of the parental single-knockout mutants, the colony morphology of double-knockout mutants showed the converged phenotype. However, no sectorization was observed in these double-knockout mutants with mutation of the *CpDmt1* gene from TdDMT2, implying that the presence of a functional *CpDmt2* gene is necessary for sporadic sectorization of TdDMT1 (Fig. 2A, bottom row).

Next, conidium production was investigated (Fig. 2B). TdDMT1 showed slightly but significantly increased numbers of conidia per plate compared to the wild type. Considering the retarded colonial growth (approximately 70% of the wild type) of the TdDMT1, the enhanced conidium production per plate indicated that conidium production per colonial area increased sharply with mutation of the *CpDmt1* gene. However, the sectored progeny of TdDMT1, TdDMT1-S, produced significantly fewer conidia, indicating a clear difference between TdDMT1-S and TdDMT1. Compared to the wild type, no difference in conidium production was observed in TdDMT2. The double mutants originating from TdDMT1 mutants showed conidium production similar to those of the wild type but were not as numerous as TdDMT1, while those from TdDMT2 showed no difference in conidium production compared to either their respective parental strains or the wild type. These results suggested that conidium production is under the control of the *CpDmt1* gene but that the presence of a functional *CpDmt2* gene is necessary for the regulation of conidium production by *CpDmt1*.

Taken together, these results suggest that although the characteristics of the single-gene mutants were distinctive, our double-knockout mutants from both TdDMT1 and TdDMT2 showed convergent colony morphology, exhibiting normal mycelial growth without either sporadic sectorization or the usual sporulation.

**FIG 3:**
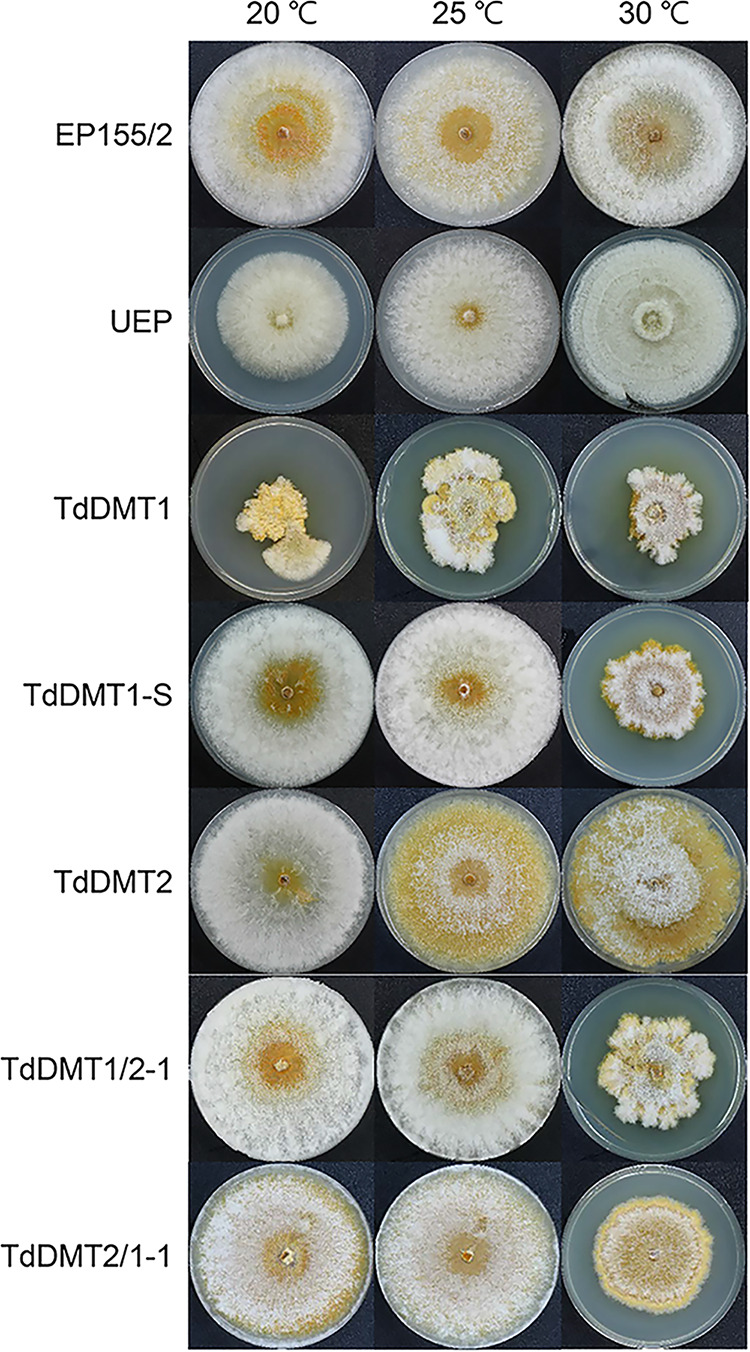
Colony morphology of DNMTase mutant strains in response to temperature stress conditions. Temperature stress was induced by incubating the plates at high (30°C) and low (20°C) temperatures relative to the standard 25°C condition.

We next measured responses to stress conditions such as high osmotic conditions, reactive oxygen species (ROS), and cell wall-inhibiting agents. When both mutants were cultured under stress conditions, no distinctive changes were observed compared to the responses of the wild type, indicating that stress responses to osmotic conditions, ROS, and cell wall-disturbing agents remained unaffected in the TdDMT1 and TdDMT2 (Fig. S2A to S2C). However, all mutants with the deleted *CpDmt1* gene, including single and double mutants, were sensitive to heat shock; incubating mutant strains at an elevated temperature of 30°C noticeably reduced their colonial growth compared to the wild type (Fig. 3). These results strongly suggest that hypersensitivity to heat stress is due solely to the mutation of the *CpDmt1* gene, independently from the *CpDmt2* gene.

10.1128/mBio.02890-20.2 FIG S2 Colony morphology of DNMTase mutant strains in response to various stress conditions. Osmotic stress (A) and oxidative stress (B) were induced by cultivation on PDAmb supplemented with sorbitol and menadione, respectively. Cell wall-disturbing stress (C) was induced using three cell wall-inhibiting agents: Calcofluor White, Congo Red, and SDS. Strains are indicated on the left, including wild-type EP155/2, hypovirulent UEP1, *CpDmt1*-null mutant (TdDMT1), a sectored progeny of TdDMT1 (TdDMT1-S), *CpDmt2*-null mutant (TdDMT2), *CpDmt2*-null mutant from TdDMT1 (TdDMT1/2-1), and *CpDmt1*-null mutant from TdDMT2 (TdDMT2/1-1). Numbers at the top of the panels (A to C) indicate the concentrations of reagents supplementing PDAmb. Download FIG S2, TIF file, 2.5 MB. Copyright © 2021 Ko et al. 2021 Ko et al. https://creativecommons.org/licenses/by/4.0/ This is an open-access article distributed under the terms of the Creative Commons Attribution 4.0 International license.

Complemented strains for the TdDMT1 and TdDMT2 showed restored wild-type phenotypes, including a lack of further sectorization, normal growth rate, and pigmentation (Fig. 2A, top row). These results clearly indicated that all observed phenotypic changes can be attributed to the corresponding DNMTase genes.

### Effect of hypovirus infection on the colony morphology of DNMTase-null mutants.

To examine the biological functions of the DNMTase genes in response to hypovirus infection, phenotypic changes were compared between CHV1-free and CHV1-infected isogenic DNMTase-null mutants. Following coculturing of the recipient DNMTase-null mutants with donor CHV1-containing UEP1, at least five putative CHV1-transferred recipient mycelium sections were independently selected and successively cultured, and single spores were isolated on hygromycin B-containing medium. The presence of hypovirus in CHV1-transferred and single-spore-isolated DNMTase-null mutant progeny was confirmed prior to further analysis.

**FIG 4:**
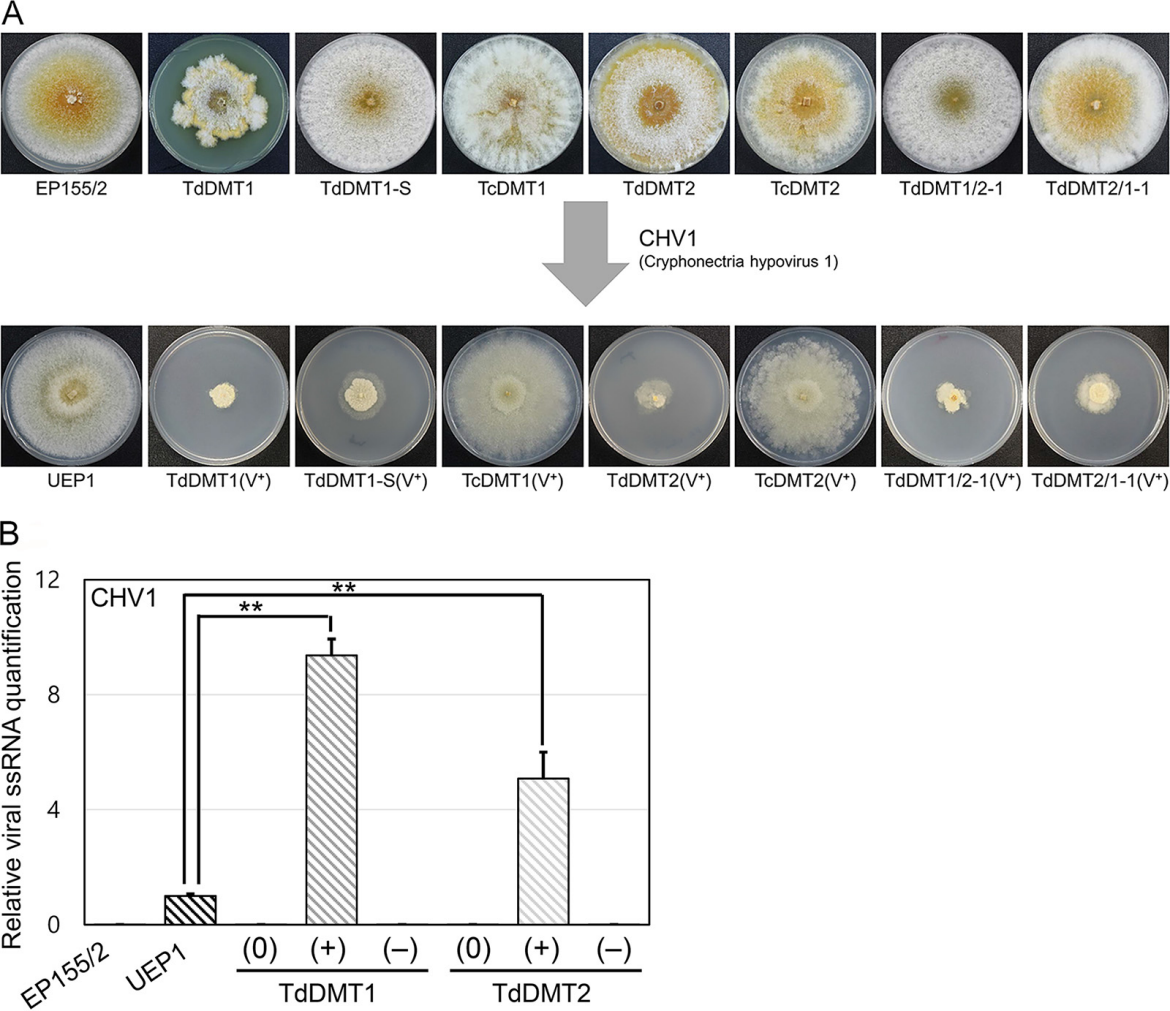
Effect of hypovirus infection on the mutant strains. (A) Colony morphology of CHV1-free (top) and CHV1-infected (bottom) mutant strains. Virus-infected isogenic strains (V^+^) are indicated. (B) qRT-PCR analysis of viral single-stranded RNA (ssRNA) accumulation. The levels of ssRNA accumulation of indicated strains are represented as the fold change relative to that of the UEP1. At least three individual experiments were performed. Values are means ± SD (error bars). Student’s *t* test was used to compare data between two groups (**, *P* < 0.01). Virus-free, virus-transferred, and cured strains are indicated by (0), (+), and (−), respectively.

Dramatic responses to hypovirus infection were observed in all mutant strains. Compared to virus-free DNMTase-null mutants, severe growth retardation was observed in all DNMTase-null mutants, including single and double mutants as well as the sectored progeny of TdDMT1, TdDMT1-S (Fig. 4A). As shown in Fig. 4A, colonial growth of all CHV1-infected DNMTase-null mutants was abnormal: colonial growth was severely retarded, aerial mycelia were not obvious, a mycelial mat on the surface of the plate was distinctive, and weak invasive growth was observed. In addition, reduced pigmentation and the near absence of conidiation were observed in both mutants.

We also transferred CHV1 to the complemented strains, which showed growth rates similar to that of UEP1, apart from severely retarded mycelial growth observed in virus-infected mutant strains. In addition, all complemented strains infected with CHV1 showed the typical virus-associated symptoms of reduced pigmentation and conidiation, as observed in UEP1. These results clearly indicate that the severely retarded growth of virus-infected null mutants was due to the absence of DNMTase genes.

We then measured the viral titer of DNMTase-null mutants using gel electrophoresis and quantitative RT-PCR (Fig. S3 and Fig. 4B). RNAs were prepared from mutants showing severely retarded colonial growth. Compared to UEP1, a significantly higher level of hypovirus titer per gram of mycelium was observed in the severely retarded mutant colonies, implying that hypoviral replication was significantly enhanced in theses colonies. These results imply that the absence of either of the two DNMTase genes results in increased accumulation of CHV1 compared to the wild type.

10.1128/mBio.02890-20.3 FIG S3 Analysis of viral titer of mutant strains using ethidium bromide-stained gel showing the viral dsRNA isolated from 0.1 g of lyophilized mycelium. The arrow indicates residual genomic DNA. Virus-free, -transferred, and cured strains are indicated by (0), (+), and (−), respectively. Download FIG S3, TIF file, 2.8 MB. Copyright © 2021 Ko et al. 2021 Ko et al. https://creativecommons.org/licenses/by/4.0/ This is an open-access article distributed under the terms of the Creative Commons Attribution 4.0 International license.

Considering the severely retarded growth, abnormal colony morphology, and higher viral titers in CHV1-infected mutants, DNMTase genes play important roles in antiviral defense, in turn suggesting that epigenetic regulation might be involved in the physiological tolerance and defense of the host fungus against hypovirus infection.

### Fungal virulence.

**FIG 5:**
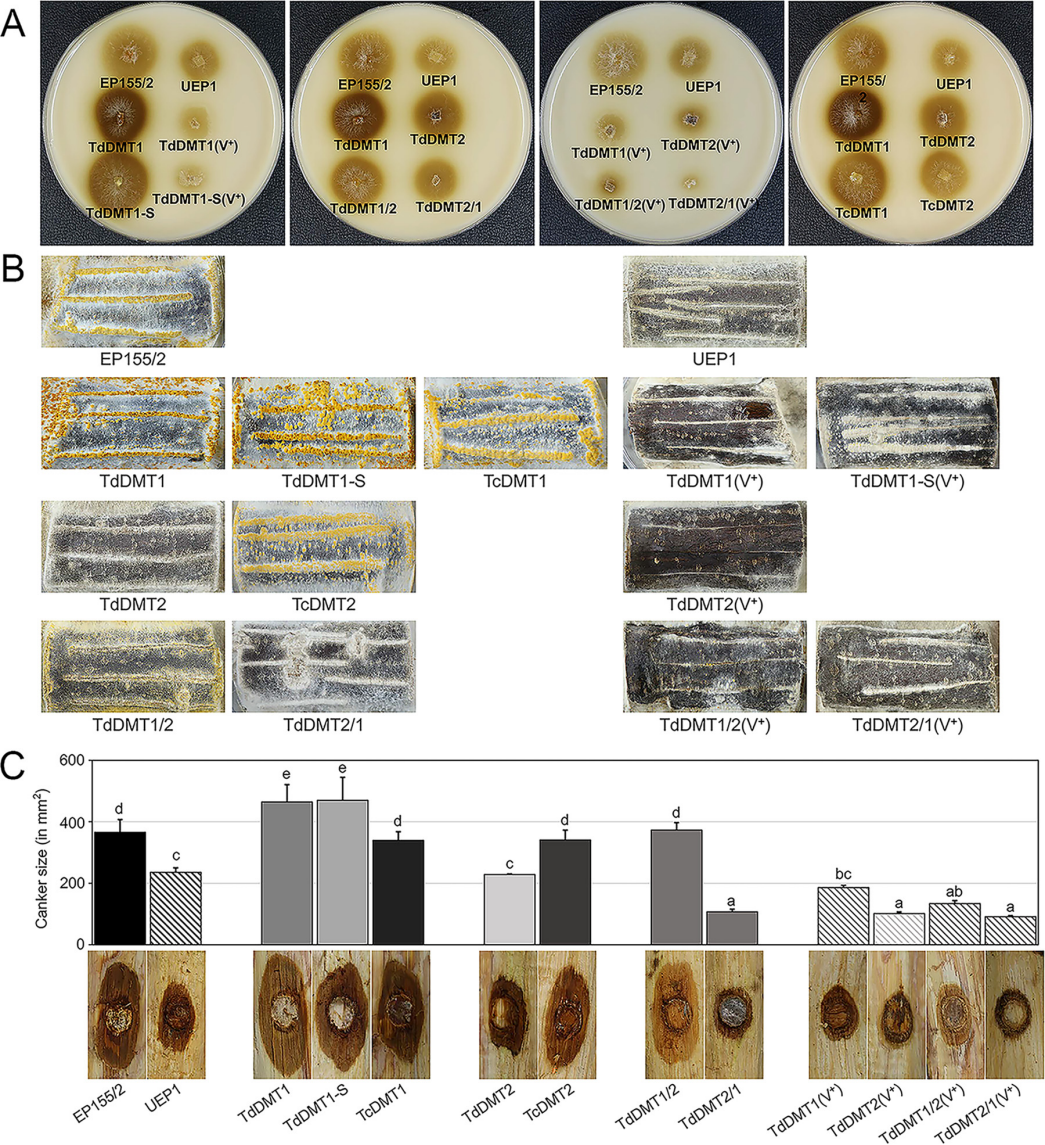
Virulence assays. (A) Bavendamm’s assay for polyphenol oxidase activity of strains. The level of brown coloration correlates with polyphenol oxidase activity of each strain. (B) Stromal pustule eruption on chestnut tree stems of strains. (C) Virulence assay using excised chestnut tree bark. Lesion measurement values are shown in square millimeters. Different letters indicate significant differences between treatments according to Duncan’s multiple range test (**, *P* < 0.01). Values are mean plus SD (error bars). DNMTase-null mutant strains containing CHV1 (V^+^) are indicated.

To measure phenol oxidation activities, we performed a Bavendamm assay, which is used as an indirect assay of fungal virulence due to its strong correlation with the virulence of *C. parasitica* (Fig. 5A). The TdDMT1 and its sectored progeny showed distinctive brown coloration around the colonies, indicative of phenol oxidase activity. These signals were more intense and larger than those of the wild type. However, the TdDMT2 produced a smaller necrotic area with diffuse brown coloration compared to the wild type. Double mutants were also examined and compared to their corresponding parental single mutants; their less intense brown coloration indicated reduced phenol oxidase activities. Regardless of strain, the virus-infected mutant strains showed very limited growth on Bavendamm medium, similar to the results on PDAmb medium. In addition, their browning was faint and not distinctive. All complemented strains showed similar levels of browning to those of the wild-type EP155/2, as expected.

Since the Bavendamm assay showed significantly increased brown coloration in the TdDMT1 but a slight decrease in browning in TdDMT2, we next examined stroma formation on the bark of chestnut tree (Fig. 5B) and the virulence of the mutant strains using excised chestnut tree bark (Fig. 5C).

The stroma formation and active pathogenic growth of mutant strains on the bark of chestnut tree was assessed by inoculating mutant strains near the excised stem (Fig. 5B). Consistent with the Bavendamm assay results, both the *CpDmt1*-null mutant and its sectored progeny produced greater numbers of distinctive stroma, which are associated with actively growing mycelia, compared to those of the wild type. In contrast, TdDMT2 showed a slightly but significantly decreased number of stroma relative to the wild type. TdDMT1/2, the double mutant originating from the single mutant TdDMT1, showed reduced pathogenic growth compared to TdDMT1. TdDMT2/1, the double mutant constructed from the single mutant TdDMT2, showed either a reduced or similar level of pathogenic growth compared to TdDMT2. The double mutants thus showed decreased pathogenic growth compared to their corresponding single mutant parents, regardless of their genetic background in terms of the parental mutant strain. Pathogenic growth was almost eliminated in all strains when infected by CHV1; in other words, an almost complete absence of normal stroma formation and active mycelial growth on the stem was observed.

We next examined the virulence of mutant strains using excised chestnut tree bark (Fig. 5C). Consistent with the Bavendamm assay and the pathogenic growth results, both the TdDMT1 mutant and its sectored progeny caused significantly larger necrotic areas on excised bark compared to the wild type. TdDMT2, by contrast, showed significantly smaller necrotic areas relative to the wild type. Likewise, the double mutants showed significantly reduced virulence compared to their corresponding single mutant parents. Almost no necrotic areas were observed around the inoculation origin in any of the CHV1-infected strains.

### Curing hypovirus from DNMTase-null mutants.

**FIG 6:**
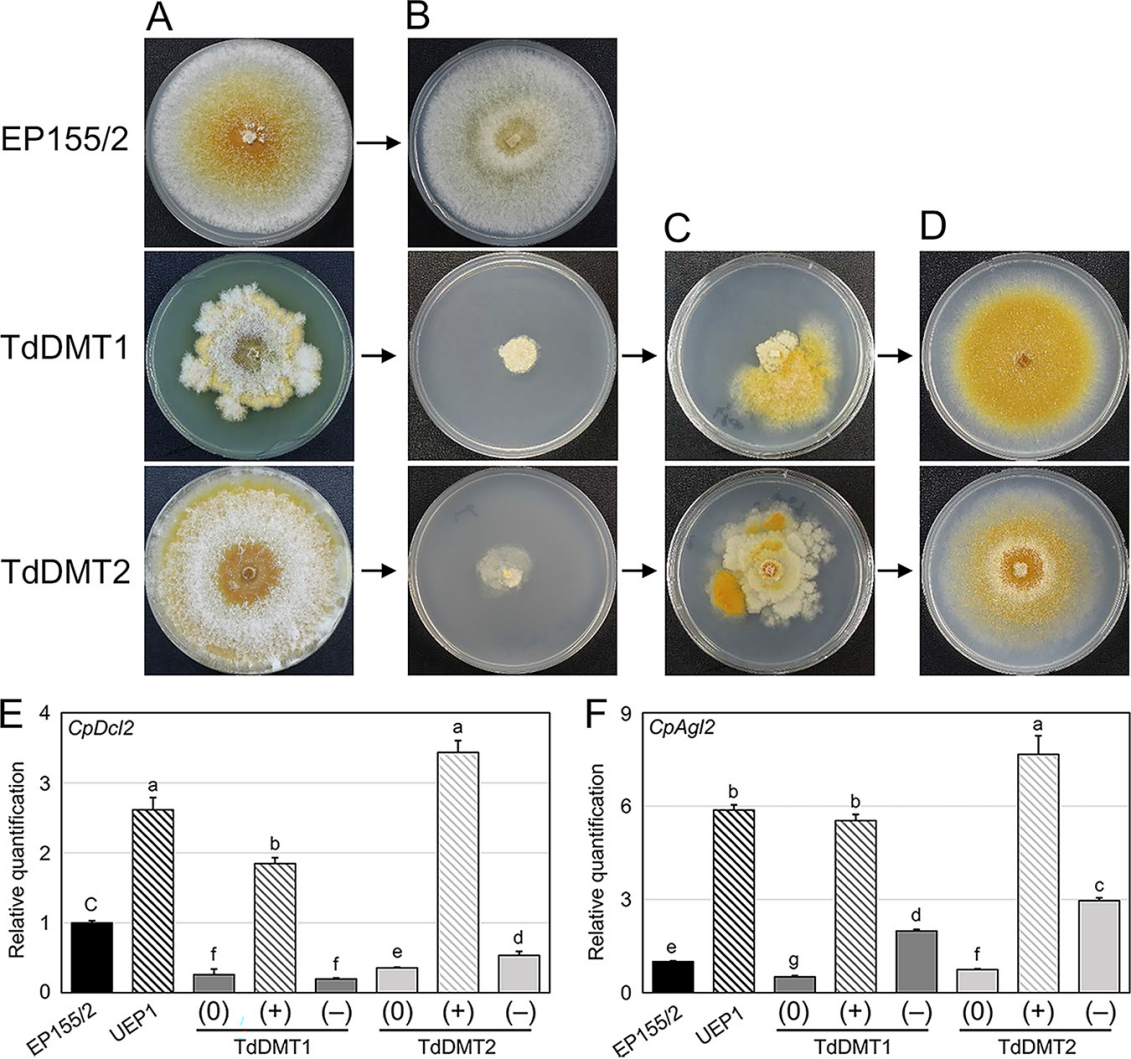
Colony morphology of CHV1-infected, curing, and cured mutant strains and expression analyses of antiviral genes *CpDcl2* and *CpAgl2*. (A) Strains indicated at the left of the panel are the wild-type EP155/2, *CpDmt1*-null mutant (TdDMT1), and the *CpDmt2*-null mutant (TdDMT2). (B) Virus-infected isogenic strains of panel A are shown, and UEP1, which is virus infected and isogenic to the wild-type EP155/2, is shown as a control. (C) Colony morphology of mutant strains showing the curing of CHV1 are represented as fast growing and well pigmented mycelial areas. (D) Colony morphology of CHV1-cured strains. (E and F) qRT-PCR analysis results for *CpDcl2* and *CpAgl2*. Changes in expression of *CpDcl2* and *CpAgl2* among mutant strains relative to the level of *gpd* are shown. Strains are indicated below the bars. Virus-free, -transferred, and cured strains are indicated by (0), (+), and (–), respectively. Different letters indicate significant differences between treatments according to Duncan’s multiple range test (**, *P* < 0.01).

Virus-infected colonies of mutants with severely retarded growth began to show distinctive mycelial growth with characteristics of the virus-free colony morphology, such as rapid growth and canonical bright yellow pigmentation (Fig. 6A to C). Virus isolation from the successively transferred progeny of these distinctive areas, followed by quantitative RT-PCR, confirmed that the hypovirus infection was no longer present in the mycelium and was effectively cured (Fig. 6B and C). This occurrence of the curing phenotype was a consistent phenomenon during cultivation of all CHV1-infected mutant strains, including single and double mutants, with more than half of the colonies showing one or more cured areas at the margin after a week of cultivation. Once the hypovirus was cured, no recurrent virus was observed in subsequent cultures of the cured strains (Fig. 6D).

To understand the molecular mechanism of hyperviral accumulation and clearance, transcriptional analyses of both the dicer-like *dcl2* and the argonaute-like *agl2* genes were conducted. This was because the *dcl2* and *agl2* genes have proved to be among various other components of the RNA silencing complex whose activities inhibit CHV1 infection in this fungus (32) and severely retarded growth after CHV1 infection was phenocopied in mutants of *dcl2* and *agl2* genes (32) (Fig. 6E and F). Compared to the wild type, accumulation of both *dcl2* and *agl2* transcripts was significantly reduced in both TdDMT1 and TdDMT2. These results strongly indicate that both of these antiviral defense genes are under the control of DNMTase genes. Significant induction of both defense genes was observed in UEP1, which is infected by CHV1 and isogenic to the wild-type strain, as previously described (32). Induction of both antiviral genes was also observed in CHV1-infected mutant strains compared to their CHV1-free counterparts. In addition, compared to fold changes in the induction levels of both the *dcl2* and *agl2* genes observed in UEP1, fold changes in the *dcl2* and *agl2* genes in the *CpDmt1*- and *CpDmt2*-null mutants were significantly greater. These results suggest that although both the *dcl2* and *agl2* genes are under the control of DNMTase genes, the absence of DNMTase genes did not affect transcriptional induction by CHV1 infection but rather increased the fold changes in the induction levels of both genes. These enhanced fold changes in antiviral genes may play a role in clearing infected CHV1 from the mutant strains. However, we believe that these antiviral defense genes are not solely responsible for viral clearance and that a more complex mechanism for this exists, since the increased amount of transcripts of both genes in TdDMT1 showing viral clearance was no larger than that of the CHV1-infected wild-type UEP1. Moreover, viral clearance was no longer observed after we transferred CHV1 to the *agl2*-overexpressing strain obtained by ectopic transformation of the chimeric *agl2* gene into the wild type (Fig. S4).

10.1128/mBio.02890-20.4 FIG S4 Colony morphology of a *CpAgl2*-overexpressing transformant during and after hypoviral transfer through hyphal anastomosis. (A) Hypoviral transfers during cocultivation from CHV1-infected UEP1 to *CpAgl2*-overexpressing transformant. Note that the peripheral area shows phenotypes of CHV1 infection, which differ from parental strains with less pigmentation. (V^+^) indicates a virus-transferred strain. (B) qRT-PCR analysis results for *CpAgl2.* Changes in expression of *CpAgl2* in the overexpressed transformant relative to the level of *gpd* are shown. Values are means ± SD (error bars). Student’s *t* test was used to compare data between two groups (**, *P* < 0.01). Download FIG S4, TIF file, 2.7 MB. Copyright © 2021 Ko et al. 2021 Ko et al. https://creativecommons.org/licenses/by/4.0/ This is an open-access article distributed under the terms of the Creative Commons Attribution 4.0 International license.

**FIG 7:**
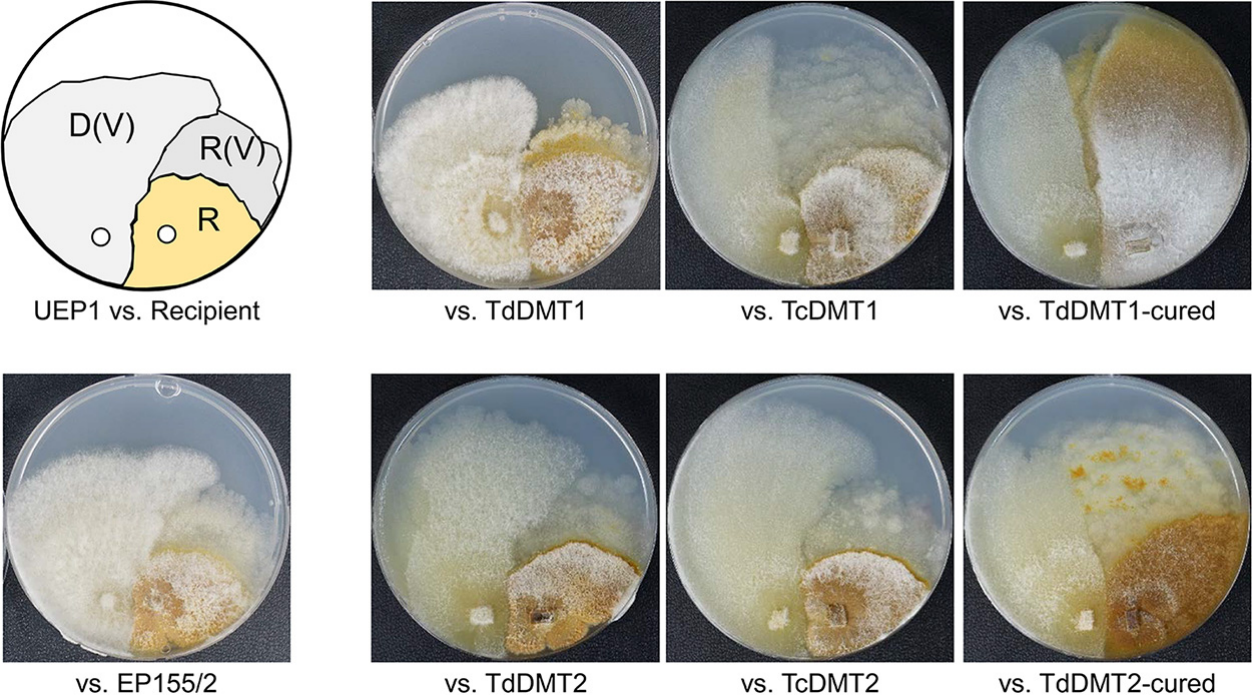
Colony morphology during hypoviral transfer through hyphal anastomosis. Hypoviral transfers during cocultivation from CHV1-infected UEP1 to strains related to *CpDmt1*-null mutation (top row) and *CpDmt2*-null mutation (bottom row) are shown. D(V), R, and R(V) indicate the virus-donor, virus-recipient, and putative virus-transferred recipient, respectively.

Considering that once CHV1 had been cleared, no further viral symptoms were observed in the area of cured mycelia during prolonged cultivation (Fig. 6D), this suggests that recurrent viral transfer from the originally infected mycelia to the cured mycelia was somehow inhibited. We thus tested whether cured strains could be reinfected by CHV1 via anastomosis. Although the ratio of successful infection to those of the parental mutant or complemented strains was not as high, reinfection of cured strains of TdDMT1 by CHV1 was possible (Fig. 7). However, characteristics of viral clearance such as distinctive pigmentation were observed directly in the growing area of the cocultured recipient cured strain of TdDMT2, suggesting that transferred CHV1 is cleared again from this reinfected cured strain. These signs of viral clearance during the viral transfer via hyphal fusion were not observed during the viral transfer to their corresponding parental mutants, which suggested that CHV1 cleared more rapidly in the cured strain than in their corresponding parental mutants (Fig. 7). In this context, it is noteworthy that significantly increased levels of *agl2* transcripts were retained in both cured mutant strains, compared to their parental virus-free null mutants and even to the wild type (Fig. 6F). Thus, with the genetic background harboring mutation of DNMTase genes, it is likely that the antiviral defense genes protect the cured strains from reinfection by CHV1 in the mixed mycelia of mutants and that these genes play a role in maintaining virus-free conditions. In addition, these molecular differences between virus-free parental mutant strains and their isogenic virus-cured mutant strains appeared to be consistent with the differences in the colony morphology of virus-free and -cured isogenic mutant strains (Fig. 6D).

## DISCUSSION

Methylation of selected cytosines in the genome sequence is the prototypic form of epigenetics in eukaryotes (33), and genome-wide reprogramming of DNA methylation has been observed during fungal development (15, 23, 34). Moreover, recent studies of the widespread and abundant phenomenon of adenine N6-methylation in fungi have emphasized the importance of DNA modification and complex control networks in genome modification (35, 36). Although DNA methylation plays important roles in diverse fungal biological processes, including gene regulation and genome defense, the specificity and biological functions of DNMTases remain unclear.

Among the various subfamilies of DNMTases, two distinct types have been found in ascomycete fungal genomes (37). The first type includes N. crassa RID (RID for repeat-induced point mutation [RIP] defective during sexual development [38, 39]) and Masc1 of Ascobolus immersus, which carries out premeiotically induced DNA methylation. The second type includes DIM-2 in N. crassa and its ortholog Masc2 in A. immersus, which are involved in all DNA methylation and gene silencing. In the *C. parasitica* genome, our earlier studies indicated that two DNMTases have evolved in the genome, with *CpDmt1* and the *CpDmt2* as apparent orthologs of *rid* and *dim-2*, respectively (15). The examination of genome sequences has provided evidence of RIP in filamentous fungi, including *C. parasitica* (40), while a recent genome analysis of *C. parasitica* revealed the presence of genome defense by RIP, at least in a few transposable elements of the genome (30). Earlier studies on the prototrophic fungal DNMTases, including RID and DIM-2, revealed that both proteins are important for genome defense in DNA methylation, but that their biological functions (e.g., vegetative or sexual development) vary across fungi (38, 39). However, other than genome defense, only a few studies have shown specific changes in development and differentiation due to mutations in fungal DNMTases, whose biological functions have been identified as follows. The balance between the formation of asexual and sexual structures has been determined by DNA methylation in N. crassa (41). Abnormal conidiation, sclerotium production, aflatoxin biosynthesis, and virulence have been observed in the *dmtA*-null mutant of Aspergillus flavus, despite a negligible level of DNA methylation (24, 42). In addition, *dmtA* has been shown as essential to sexual development in Aspergillus nidulans (43). Mutants of *rid* orthologs in the entomopathogenic fungus Metarhizium robertsii and plant-pathogenic fungus Magnaporthe oryzae have shown minor phenotypic changes, while mutants of *dim-2* orthologs in M. robertsii and M. oryzae have shown impairment of development and virulence (23, 25).

Here, in addition to sporadic sectorization, reduced colonial growth, increased conidium production, and hypersensitivity to heat shock in TdDMT1, we have described dramatic changes in virulence in TdDMT1, which showed significant enhancements of virulence and the associated phenol oxidase activity. In addition, the sectored progeny of TdDMT1 produced significantly larger necrotic areas on inoculated excised chestnut tree bark, as well as higher enzyme activity. These results show that in both sectored progeny and parental strains, the virulence of strains with the *CpDmt1* mutation was affected in the same direction, implying that even though virulence and sectorization are under the control of the *CpDmt1* gene, each pathway is modulated independently by different sets of control genes. Our recent study has shown significant changes in the secondary metabolites of sectored strains, which were accompanied by global changes in DNA methylation (8). In addition, numerous studies have shown that fungal secondary metabolites are affected by treatment with epigenetic modifying agents or inhibitors such as 5-azacytidine (44–47). Although fungal secondary metabolites are complex and their biological functions remain unclear, they are assumed to affect adaptation to their specific ecological niche, such as successful colonization of a specific host (48). Thus, considering that both parental TdDMT1 and their sectored progeny have shown similar levels of increased virulence, specific sets of secondary metabolites are involved in virulence and the sectored phenotype. Although only slight changes in colony morphology were observed in TdDMT2, it showed significantly reduced virulence. These results of characteristic changes in virulence caused by the two DNMTases, albeit in opposite directions, have confirmed that *CpDmt1* and *CpDmt2* genes are not functionally redundant but play specific roles, with the biological function of *CpDmt1* differing from that of *CpDmt2*. Considering the results for *M. robertsii* and *M. oryzae*, where *dim-2* orthologs rather than *rid* orthologs showed severe phenotypic changes, our results differed markedly, with *CpDmt2* as well as *CpDmt1* gene driving distinctive phenotypic changes. Thus, the characteristics of functional specificity differed between fungi. Although the characteristics of the single-gene mutants were distinctive, our double-knockout mutants from both TdDMT1 and TdDMT2 showed convergent phenotypic changes, exhibiting recovered mycelial growth with normal sporulation. However, our double mutants were not identical but showed difference in virulence. Compared to their corresponding single mutant parents, double mutants showed reduced virulence; that is, the double mutant originating from TdDMT1 showed reduced virulence compared to its parental strain, whereas the double mutant from TdDMT2 did not show the increased virulence of its parental strain. Although further studies are required to explain these differences, these results imply that difference in the expression of target genes, depending on the sequential order of mutations, might be characteristics of the epigenetic control of fungal genes.

Our results clearly indicated that both DNMTases in *C. parasitica* affected fungal development by causing sporadic sectorization and virulence. However, we observed no significant changes in response to the tested stressors with mutation of the DNMTases, implying that DNA methylation affects a broad range of very specific pathways in this fungus.

The effects of hypoviral CHV1 infection on the mutant strains were so dramatic that all infected strains, including both single and double mutants, showed retarded growth on plates. Moreover, significant increases in the CHV1 titer were observed in CHV1-infected mutants with severe growth retardation. Similarly dramatic growth inhibition due to CHV1 infection was observed in *dcl2* and *agl2* mutants (32). This severe impact on the growth of CHV1-infected DNMTase mutants appeared to be due not simply to the higher level of CHV1 titer in host cells, since CHV1-infected *dcl2* and *agl2* mutants showed similar growth retardation but no change in CHV1 titer (32). Recent studies on the Stp-Ada-Gcn5 acetyltransferase (SAGA) complex revealed the importance of SAGA in antiviral as well as host response (49, 50). Unlike the mutants of the SAGA component, our mutants showed no changes in the induction of antiviral genes such as *dcl2* and *agl2* by CHV1 infection. Further studies, however, will be required to determine whether the pathways that regulate the host response gene overlap. Overall, these results indicate that the functions of DNMTases play an important role in defending fungal growth and development against hypovirus infection.

Although the stability of vertical transmission (from vegetative to regenerative parts) of mycoviruses varied between fungi and the uneven distribution of mycoviruses within a single colony of *C. parasitica* (51) and unstable maintenance of a mycoreovirus during subculturing of *C. parasitica* (52) have all been acknowledged, the stable persistence of CHV1-EP713 within vegetative parts such as the mycelium of *C. parasitica* has been widely accepted (1, 2). Thus, the spontaneous virus clearance observed in colonies of CHV1-infected TdDMT1 and TdDMT2 is exceptional and has never previously been reported in these fungus-virus interactions. It is interesting to note that loss-of-function mutants of DNMTase genes, which function as factors in modulating fungal growth and defense against hypovirus infection, showed spontaneous curing of viral infection. Thus, a more complex mechanism of viral clearance appears likely, as evidenced by the considerable viral titers in CHV1-infected mutant strains with retarded colonial growth that nonetheless consistently cleared their viral infections. Once viral infection was cured, no reinfection of CHV1 from the older mycelial region (virus infected with severely retarded growth) was observed based on the colony morphology of the virus-free region; the virus-cured area showed normal mycelial growth, pigmentation, sporulation, and no further symptoms of CHV1 infection. Considering the continuity of fungal cytoplasm, these results strongly suggest that viral clearance occurs not through a passive mechanism such as viral escape due to an uneven distribution of infected CHV1 but rather via an unknown mechanism that eliminates the virus from infected cells. These results also suggest a lack of cytoplasmic continuity between the CHV1-infected and cured regions. Taken together, our results clearly indicate that DNMTase genes play an important role in the initial host response to viral infection, including fungal growth and development, and in viral propagation, as well as for later fungal responses to viral infection, such as viral clearance. In this context, our observation of spontaneous viral clearance due to the absence of DNMTase genes is of particular interest. Considering that in our earlier study that TdDMT1 and TdDMT2 mutants showed various degrees of DNA methylation and specifically, that TdDMT2 showed dramatically decreased DNA methylation while TdDMT1 showed only slight change (15), the underlying mechanism for viral clearance might be a biological function unique to DNMTases, a function other than DNA methylation. Thus, further investigation of this phenomenon may clarify how viral infection is cured and reinfection is inhibited in other systems.

## MATERIALS AND METHODS

### Fungal strains and cultivation.

The wild-type *C. parasitica* strain EP155/2 (ATCC 38755) and its isogenic CHV1-EP713-infected strain UEP1 were maintained on PDAmb plates containing potato dextrose agar (PDA) supplemented with l-methionine (0.1 g/liter) and biotin (1 mg/liter) (PDAmb) and kept at 25°C with constant low light (53, 54). Single-knockout mutants of *CpDmt1* (TdDMT1) and *CpDmt2* (TdDMT2), as well as their corresponding complemented strains (TcDMT1 and TcDMT2, respectively) were obtained from a previous study (15). Fungal cultivation conditions and methods for preparing the primary inoculum for liquid cultures were similar to those described previously (53, 55). For spore counting, strains were cultured for 3 weeks at 25°C on PDAmb medium to obtain spores. The number of spores in each plate harvested with 10 ml of sterile distilled water was determined using a hemocytometer (53). The harvested mycelia were stored at −70°C and lyophilized to preserve intact DNA and RNA as described previously (55).

### RNA extraction, cDNA synthesis, and quantitative real-time reverse transcription-PCR (qRT-PCR).

Total RNA was extracted as described previously (53). To quantify the expression levels of target genes, equal amounts of cDNAs were synthesized using 0.5 μg total RNA treated with RQ1 RNase-free DNase I (Promega, Madison, WI, USA), SuperScript IV reverse transcriptase (Thermo Fisher Scientific, Waltham, MA, USA), and oligo(dT) according to the manufacturer’s protocols. qRT-PCR was performed using AmpiGene qPCR Green Mix Lo-ROX (Enzo Biochem, New York, NY, USA) and assessed with a GeneAmp 7500 sequence detection system (Applied Biosystems, Foster City, CA, USA), as described previously (56). Viral accumulation was analyzed as described previously (57). The glyceraldehyde-3-phosphate dehydrogenase gene (*gpd*) was used as an internal control. Analyses were conducted from at least two independent RNA preparations, in triplicate for each transcript, using primers specific to *gpd* and the target genes. Primer pairs for each gene are provided in Table S1 in the supplemental material (15). Transcript abundance, relative to the amount of *gpd*, in each sample was calculated based on fold change in the expression of target genes, normalized to the internal control *gpd* (58).

10.1128/mBio.02890-20.5 TABLE S1 List of PCR primer sequences. Download Table S1, PDF file, 0.07 MB. Copyright © 2021 Ko et al. 2021 Ko et al. https://creativecommons.org/licenses/by/4.0/ This is an open-access article distributed under the terms of the Creative Commons Attribution 4.0 International license.

### Construction of a replacement vector and fungal transformation.

To construct the double-deletion mutant, TdDMT1/2 and TdDMT2/1, *CpDmt2* and *CpDmt1* were deleted from TdDMT1 and TdDMT2, respectively. We employed the same strategy used for single deletions, except that instead of using the hygromycin B gene cassette (*hph*) for selection, the Geneticin resistance cassette (*G418*) was amplified from the pBSSKG plasmid harboring *G418* (59) using the primers shown in Table S1. The replacement vectors pDGDMT1 and pDGDMT2, which were constructed by fusing *G418* with the 5′ and 3′ flanking regions of *CpDmt1* and *CpDmt2*, respectively, were cloned into the pGEM T-easy vector and then used for transformation into the virus-free wild-type EP155/2 strain.

Protoplast preparation and transformation of *C. parasitica* were performed as described previously (5, 53). Transformants were selected from PDAmb plates supplemented with 150 μg/ml Geneticin (Invitrogen, Carlsbad, CA, USA.) and passaged three or four times on selective medium, and then single spores were isolated as described previously (60). PCR and Southern blot analyses were conducted with genomic DNA from the transformants to confirm replacement of the *CpDmt1* and *CpDmt2* genes.

For overexpression of the *agl2* gene, chimeric structures of the *agl2* gene were constructed using the genomic DNA clone of the *agl2* gene (GenBank accession no. GQ250185.1) and the constitutive expression cassette (61). The resulting vector was then used to transform the wild-type EP155/2. Transformants were selected from agar plates supplemented with 150 μg/ml hygromycin B (Calbiochem, San Diego, CA, USA), and single-spored stable transformants were further analyzed for the expression of the *agl2* gene using qRT-PCR.

### Southern blot analysis.

Genomic DNA from *C. parasitica* was extracted as described previously (5). The genomic DNA (20 μg) was digested with the restriction enzymes HindIII and XhoI, blotted onto nylon membranes, and hybridized with radioactively labeled probes.

### Responses of DNMT-null mutants to stressors.

The responses of DNMT-null mutants to various stressors were compared with those of the wild-type EP155/2 and its isogenic hypovirulent UEP1 strains. PDAmb medium was supplemented with four different concentrations of sorbitol (0.25, 0.5, 1.0, and 2.0 M) (56), menadione (25, 50, 75, and 100 μM) (62), and three cell wall-disrupting agents (Congo Red [CR], sodium dodecyl sulfate [SDS], and Calcofluor White [CFW]) (63) to investigate the strains’ responses to osmotic pressure, ROS, and cell wall-disrupting agents, respectively.

### Virulence assays.

In order to analyze the pathogenicity of the strains by measuring phenol oxidase activity, the strains were cultured on Bavendamm’s medium containing 0.5% tannic acid (31). Stromal pustule production on chestnut stems was measured by inoculating the agar block containing actively growing mycelia from respective strains inoculated into each side of sterile pieces of chestnut stems that were artificially wounded for 4 weeks (64). Virulence assay using excised chestnut trees were performed as described previously (65). Three replicates for each strain were used, and each experiment was repeated twice.

### Transmission of CHV1.

Virus transmission was performed as described previously (66). Briefly, mycelial plugs of the virus-containing strain UEP1 were placed on PDAmb medium adjacent to mycelial plugs of virus-free recipient transformants. After 7 days of cocultivation, putatively fused mycelia along the border between each pair of strains were transferred to hygromycin-containing PDAmb and examined for the presence of a sector with differing colonial phenotypes, such as reduced growth or pigmentation. Mycelia in such sectors were successively transferred to fresh hygromycin-containing medium, and the strains were single spore isolated to select for virus-infected recipient transformants. The presence of hypovirus was confirmed through purification of double-stranded RNA (dsRNA) from single-spore isolates.

### Isolation of dsRNA from *C. parasitica*.

dsRNA was isolated according to a procedure described previously (67). UEP1 and CHV1-transmitted transformants were grown on cellophane membranes overlaying PDAmb for 7 days at 25°C. Nucleic acid was extracted using 1 ml of extraction buffer (2× STE [0.2 M NaCl, 0.1 M Tris-HCl {pH 8.0}, 2 mM EDTA], 2% SDS, 1% sodium bisulfate) with 0.1 g lyophilized mycelium. Following two successive phenol extractions, dsRNA was isolated using cellulose (Sigma-Aldrich, St. Louis, MO, USA) and analyzed via electrophoresis in a 0.8% agarose gel.

### Statistical analysis.

Statistical assessments were performed using SPSS software (version 23.0; SPSS Inc., Chicago, IL). All data for qRT-PCR transcripts and canker areas were statistically evaluated using analysis of variance (ANOVA) and Student’s *t* test. The significance of the effects was determined using Duncan’s multiple-range test at *P* < 0.01.
