# Supplementary figures and images for: Distinct Roles of Two DNA Methyltransferases from Cryphonectria parasitica in Fungal Virulence, Responses to Hypovirus Infection, and Viral Clearance
Source: mBio. 2021 Feb 9;12(1):e02890-20. doi: 10.1128/mBio.02890-20 (PMC8545091; doi:10.1128/mBio.02890-20)

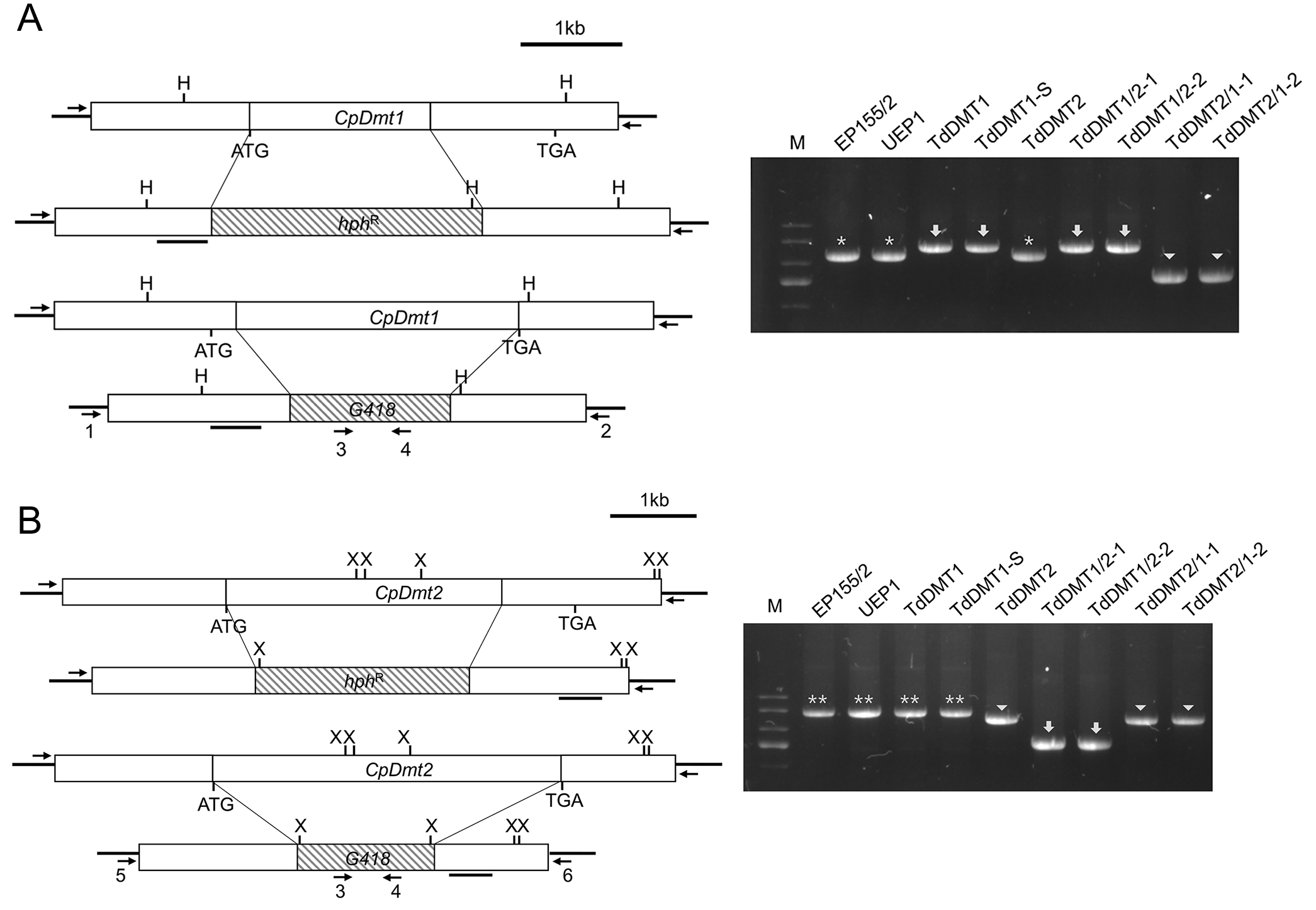

Supplement: FIG S1 [file mbio.02890-20-sf001.tif]

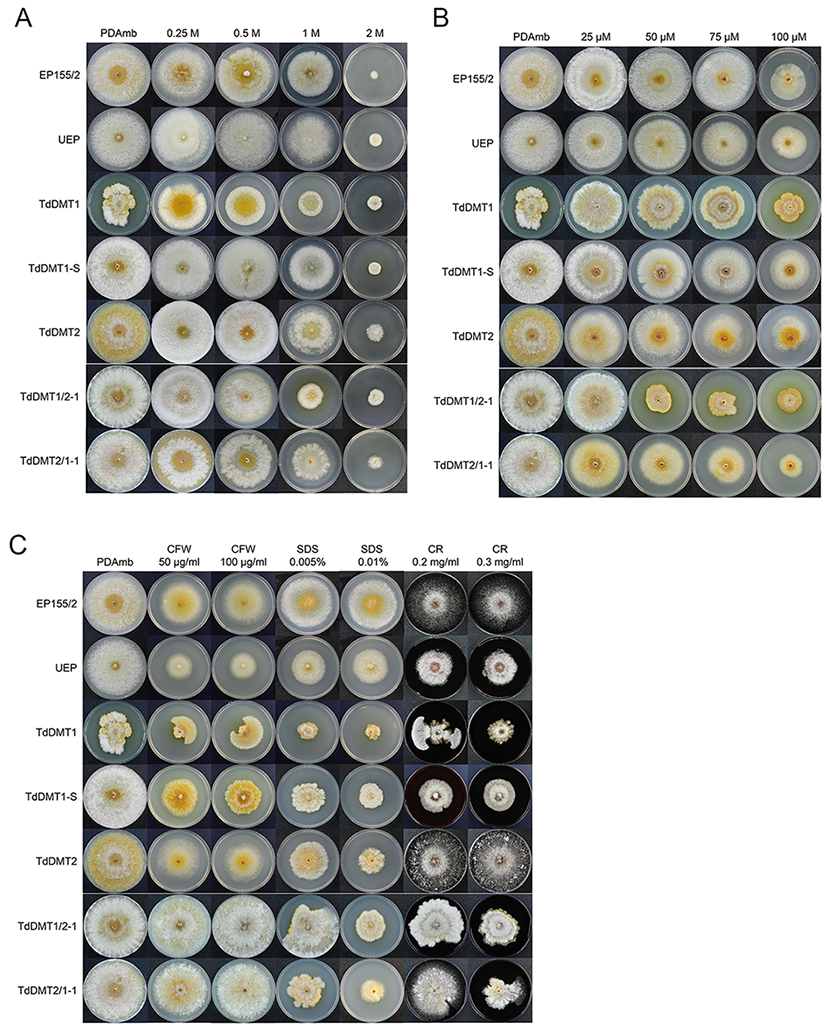

Supplement: FIG S2 [file mbio.02890-20-sf002.tif]

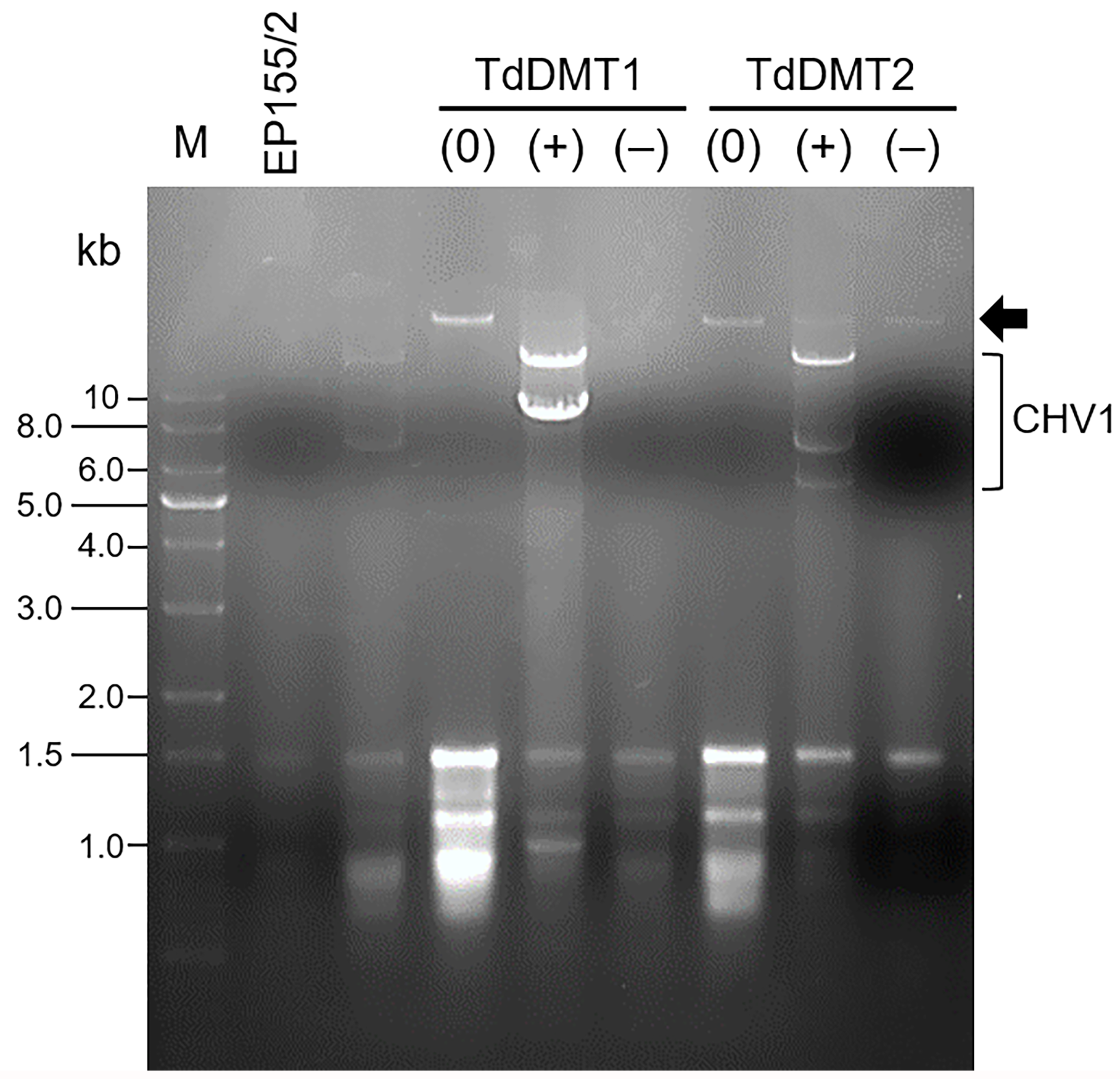

Supplement: FIG S3 [file mbio.02890-20-sf003.tif]

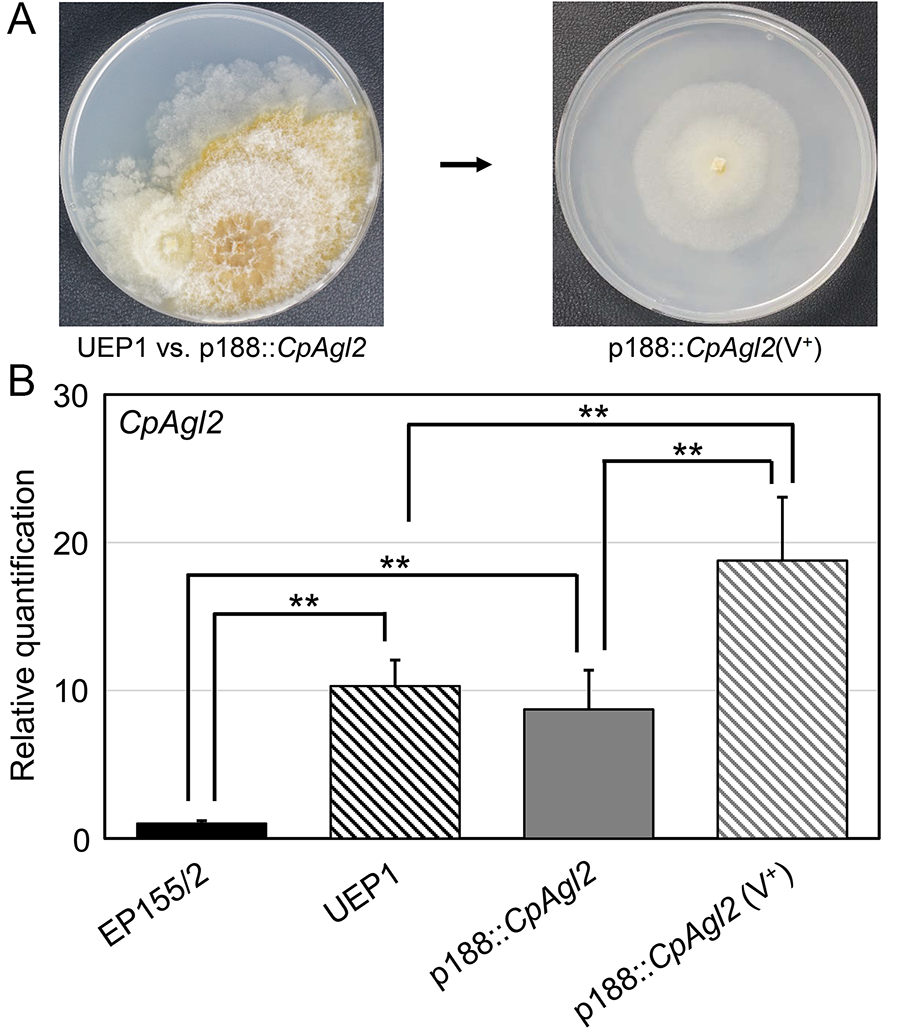

Supplement: FIG S4 [file mbio.02890-20-sf004.tif]
