# Supplementary material for: Distinct Roles of Two DNA Methyltransferases from Cryphonectria parasitica in Fungal Virulence, Responses to Hypovirus Infection, and Viral Clearance
Source: mBio. 2021 Feb 9;12(1):e02890-20. doi: 10.1128/mBio.02890-20 (PMC8545091; doi:10.1128/mBio.02890-20)
Supplement: TABLE S1 [file mbio.02890-20-st001.pdf]

Table S1 List of PCR primer sequences.

Primer	Name	Primer Sequence (5’-3’)	Use
1	TdDMT1-F	GCACCAAAATTTCTCACCCC	Screening of mutant
2	TdDMT1-R	CCTTTCCCTGCCCAACAACT	Screening of mutant
3	G418-F	GGCTATCGTGGCTGGCCA	Screening of mutant
4	G418-R	GCGAATCGGGAGCGGCGA	Screening of mutant
5	TdDMT2-F	GCAGTGACGGATCTCACCAA	Screening of mutant
6	TdDMT2-R	GGAAGGCCTCCCAGGTCTCC	Screening of mutant
7	CpDmt1_G-F	AGGGCACCAGGAACCCTTGC<u>CTCGAGGACGTTAACTGATATTGAAGG</u>	Construction of *CpDmt1*-null mutant. Underline: indicates the geneticin resistant cassette (*G418*) part
8	CpDmt1_G-R	<u>TCCTTCAATATCAGTTAACGTCCTCGAG</u>GCAAGGGTTCCTGGTGCCCT	Construction of *CpDmt1*-null mutant. Underline indicates the *G418* part
9	G_CpDmt1-F	<u>GTTCTCGAGGTCGACGGTATCG</u>GTGCGACTTGCTGGAGAAAAGT	Construction of *CpDmt1*-null mutant. Underline indicates the *G418* part
10	G_CpDmt1-R	ACTTTTCTCCAGCAAGTCGCAC<u>CGATACCGTCGACCTCGAGAAC</u>	Construction of *CpDmt1*-null mutant. Underline indicates the *G418* part
11	CpDmt2_G-F	CTCAGACTTTGCGCTATT<u>CTCGAGGACGTTAACTGA</u>	Construction of *CpDmt2*-null mutant. Underline indicates the *G418* part
12	CpDmt2_G-R	<u>TCAGTTAACGTCCTCGAG</u>AATAGCGCAAAGTCTGAG	Construction of *CpDmt2*-null mutant. Underline indicates the *G418* part
13	G_CpDmt2-F	<u>TCGAGGTCGACGGTATCG</u>AAGATCCCGCCCGTCGCA	Construction of *CpDmt2*-null mutant. Underline indicates the *G418* part
14	G_CpDmt2-R	TGCGACGGGCGGGATCTT<u>CGATACCGTCGACCTCGA</u>	Construction of *CpDmt2*-null mutant. Underline indicates the *G418* part
